# Prevalence, Tetracycline Resistance and Tet(O) Gene Identification in Pathogenic *Campylobacter* Strains Isolated from Chickens in Retail Markets of Lima, Peru

**DOI:** 10.3390/antibiotics11111580

**Published:** 2022-11-09

**Authors:** Christian Benites, Diego Anampa, Domingo Torres, Ivette Avalos, Miguel Rojas, Carlos Conte, César Lázaro

**Affiliations:** 1 Laboratorio de Farmacología y Toxicología Veterinaria, Facultad de Medicina Veterinaria, Universidad Nacional Mayor de San Marcos, Lima, Apartado 03-5137, Peru; 2 Laboratorio de Inmunología, Facultad de Medicina Veterinaria, Universidad Nacional Mayor de San Marcos, Lima, Apartado 03-5137, Peru; 3 Instituto de Química, Universidade Federal do Rio de Janeiro, Av. Athos da Silveira Ramos, 149, Cidade Universitária, Rio de Janeiro 21941-909, Brazil

**Keywords:** antibiotic resistance, *Campylobacter*, chicken, tetracyclines

## Abstract

Background: In this study, we aimed to estimate the prevalence, tetracycline resistance and presence of Tet(O) in *Campylobacter* strains isolated from chicken in markets of Lima, Peru. Methods: A total of 250 chicken samples were obtained from traditional markets (skin, *n* = 120) and supermarkets (meat, *n* = 130). Samples were subjected to microbiological assays for identification of *Campylobacter* spp. according to ISO 10272-2017, and the isolates were then submitted to species identification by PCR. Phenotypic resistance to tetracyclines was assessed by the Kirby–Bauer test, and the presence of the Tet(O) gene was determined by PCR. Results: A significantly higher prevalence (*p* < 0.0001) of *Campylobacter coli* in skin samples from traditional markets (97.5%) than in meat samples from supermarkets (36.2%) was observed. On the other hand, *Campylobacter jejuni* was confirmed only in 3.1% of meat samples. All *Campylobacter* species isolated from skin and meat samples were phenotypically resistant to tetracyclines; however, the presence of the Tet(O) gene in *C. coli* was identified in 76.9% and 66.0% of skin and meat samples, no significant statistical difference (*p* = 0.1488) was found between these prevalence. All *C. jejuni* isolated from chicken meat samples from supermarkets were positive for Tet(O) gene. Conclusions: This study confirms the high prevalence of *C. coli* isolated from chicken sold in traditional markets and supermarkets in Lima, Peru, and in more than 70% of these strains, phenotypic resistance to tetracyclines could be linked with expression of the Tet(O) gene. It is necessary to evaluate other genes involved in resistance to tetracyclines and other groups of antibiotics in campylobacter strains isolated from chicken meat.

## 1. Introduction

Pathogenic species of *Campylobacter* are involved in not only human diseases, such as gastrointestinal discomfort, but also autoimmune diseases, such as Guillain–Barre and Miller Fisher syndromes [1,2]. *Campylobacter jejuni* and *Campylobacter coli* are the most important species associated with campylobacteriosis, an infectious disease associated with vomiting, diarrhea, abdominal pain, and fever that is considered a potential public health risk [3]. *Campylobacter* infections in humans are frequently self-limiting; however, when signs of diarrhea and fever persist, especially in children, the elderly, and immunocompromised people, antibiotics such as fluoroquinolones, aminoglycosides, tetracycline, and macrolides are prescribed [4].

The use of antibiotics in animals for human consumption is one of the reasons for the increment of antibiotic resistance in bacterial strains [5]. Tetracyclines are an antibiotic group used in human and veterinary medicine. These drugs act by inhibiting protein synthesis by interrupting the binding of aminoacyl-tRNA to the acceptor site in the mRNA–ribosome complex of the 30S ribosome subunit. Resistance to this group is determined by the presence of the Tet(O) gene, which expressed a ribosome protection protein, by promoting the release of the drug from its site of inhibition on the ribosome [6,7]. The annual report on antimicrobial agents intended for use in animals developed by the World Organization for Animal Health (WOAH) in 2022 indicates that poultry farming is a major agricultural sector where antibiotics are used, and tetracyclines represent the group most frequently used in farm animals, which applies not only to the Americas but also to Africa, Asia, the Far East, Oceania, and Europe [8]. In general, chickens are considered carriers and transmitters of bacteria that may contain various antimicrobial resistance genes [9].

*Campylobacter* spp. can survive in chickens, e.g., in part of the gut microbiota, and their presence is not related to changes in chicken cecal mucosae. The presence of this microorganism in chicken and chicken products can be attributed to several factors; however, a critical point for determining transmission to humans is related to deficient slaughter practices, where chicken meat could be contaminated by the gut content containing *Campylobacter* spp. [10,11,12]. In Peru, as in other developing countries, the chicken slaughter process is carried out manually or semi-technically, both of which apply to the national commercialization of chicken meat in traditional markets and supermarkets. The traditional market represents around 80% of the entire Peruvian poultry distribution chain, promoted by its low cost, informal business, and lack of official control [13].

*Campylobacter* spp. in chicken meat is a pre-existing health problem further exacerbated by the presence of strains of this microorganism that have developed antimicrobial resistance. The presence of tetracycline-resistant *Campylobacter* strains has been reported in different parts of the world, including Europe [14], Southeast Asia [15], and Africa [16]. Such reports highlight the variability in *Campylobacter* prevalence and the increase in antibiotic resistance. In Peru, previous reports showed the presence of *C. jejuni* and *C. coli* in chicken meat from small-scale slaughter and traditional markets with the presence of resistance to macrolides related to the ermB gene [17,18]. However, the information on the resistance of pathogenic *Campylobacter* species to other groups of antibiotics should be considered when conducting a national overview toward implementing mechanisms for prevention and control of infection. This study aims to determine the prevalence, phenotypic tetracycline resistance, and the presence of the Tet(O) gene in *Campylobacter* strains isolated from retail markets in Lima, Peru.

## 2. Results

According to microbiological assay, 173/250 (69.2%) of samples presented strains corresponding to *Campylobacter* spp.; of these, the hippurate test identified 169 strains of *C. coli* and only 4 as *C. jejuni*. Subsequent molecular analyses confirmed that 164 strains were *C. coli*, and 4 were *C. jejuni*. The PCR assay of chicken skin samples from traditional markets led to the identification of 117/120 (97.5%) strains as *C. coli* and none as *C. jejuni*. On the other hand, of the 130 chicken meat samples from supermarkets, 4 (3.1%) and 47 (36.2%) were identified as *C. jejuni* and *C. coli*, respectively. Overall, pathogenic *Campylobacter* was isolated and confirmed in 168 chicken samples: 117 from the skin (traditional markets) and 51 from meat (supermarkets). The high prevalence of *C. coli* in skin samples compared with meat samples was statistically significant (*p* < 0.05) by the Chi-square test (Table 1). Three positive *C. jejuni* strains were subjected to sequencing corresponding to the hipO gene (735 bp), it is responsible for the hippurate activity exclusively found in *C. jejuni*. These sequences were deposited to GenBank with accession codes ranging from OP503634 to OP503636.

**Figure 1:**
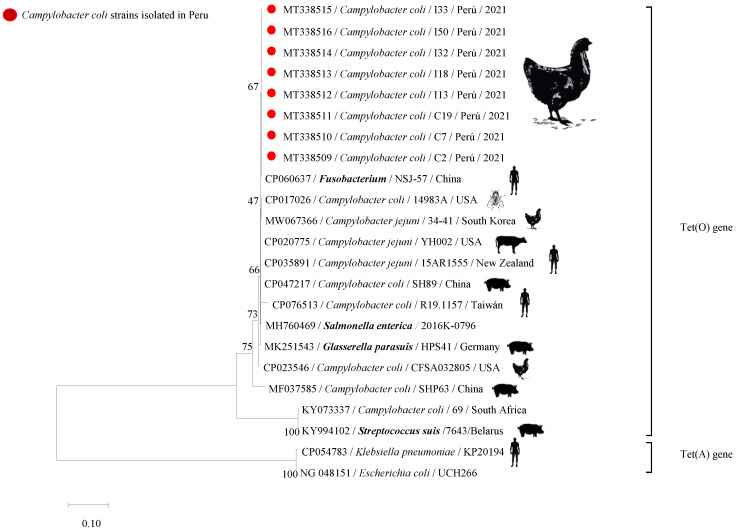
Phylogenetic tree constructed from nucleotide sequences (559 bp) of the Tet(O) gene from *Campylobacter coli* strains. Obtained sequences were aligned and compared with sequences of the genes encoding the Tet(O) and Tet(A) proteins expressed in different genera and species of bacteria isolated from different animal species. The genetic region used for detecting Tet(O) was found to be highly conserved among those reported for different genera and species, with a phylogenetic distance in the range of 77.9% to 100% nucleotide identity. When aligning Tet(O) and Tet(A) sequences, a nucleotide identity of between 41% and 43.6% is observed. Calculated phylogenetic distances were corrected using the parameter Kimura-2 model. The dendrogram was performed with the neighbor-joining method. Statistical support was performed by bootstrapping of 1000 replicates. Bootstrap values greater than 75% occur at nodes and branches. The distance scale is in substitutions/site. The red circles indicate the Peruvian strains of *Campylobacter coli* that express the Tet(O) gene. The eight analyzed sequences were submitted to GenBank with the accession numbers MT338509 to MT338516.

The Kirby–Bauer test showed a phenotypic antibiotic resistance for doxycycline and tetracycline in 100% of the strains of the previously identified *C. coli* (164/164) and *C. jejuni* (4/4). According to CLSI, a zone diameter breakpoint less than 26 mm indicates the resistance of *Campylobacter* strains. Although all isolated *C. coli* and *C. jejuni* strains showed resistance to both types of antibiotics, it is important to highlight that some strains expressed different inhibition diameters for tetracycline (ranging from 8 to 12 mm) and doxycycline (ranging from 10 to 14 mm), and some strains did not show an inhibition zone (Supplementary Tables S1 and S2). The PCR assay confirmed the presence of the Tet(O) gene in a total of 121/164 (73.8%) strains of *C. coli* (Supplementary Figure S1). The presence of the Tet(O) gene was only found in 90 strains of *C. coli* (76.9%) isolated in chicken skin samples from traditional markets. On the other hand, 4/4 *C. jejuni* (100%) and 31/47 *C. coli* (66.0%) strains isolated in chicken meat samples from supermarkets were confirmed to harbor the Tet(O) gene. The Cohen’s Kappa index equal to 0.656 (CI between 0.568 to 0.744) showed a substantial agreement between phenotypic and genotypic results. Although the prevalence of tetracycline resistance *C. coli* with Tet(O) gene at the skin level (Traditional markets) was higher than in meat (Supermarkets), no significant statistical difference (*p* = 0.1488) was observed. Eight sequences of 559 bp subjected to sequencing corresponded to Tet(O). These sequences were deposited to GenBank with accession codes ranging from MT338509 to MT338516. Phylogenetic analysis of these sequences is presented in tree form in Figure 1.

## 3. Discussion

Our results showed the presence of pathogenic *Campylobacter* in more than 50% of chicken product samples. These results indicate a high prevalence of *Campylobacter* isolated in chicken from retail markets in Lima, Peru. According to previous reports, Lucas et al. [19] found that 20% of *Campylobacter* spp. in chicken carcasses and cecal content were from unauthorized slaughterhouses. Lázaro et al. [18] found that 21.1% of *Campylobacter* spp. in carcass (eviscerated and non-eviscerated) and cecal content of chicken from small-scale slaughterhouses. It is appropriate to highlight the differences in the presence of *Campylobacter* spp. found in traditional markets was 117/120 (97.5%) compared to supermarkets with 56/130 (43.1%). In Peru, similarly to other countries in South America, chicken meat is sold in traditional markets, where the slaughter process is performed in inappropriate facilities near markets known as “peladurias.” This type of procedure is common in almost all traditional markets of Peru. It has remained due to the Peruvian consumers themselves since they associate the freshness of the chicken meat with freshly slaughtered chickens. However, another critical factor is the product cost, where the price is lower in traditional markets than in supermarkets. In traditional markets, the contamination of chicken meat is promoted by the conditions of the “peladurias” and the market stands. Both have poor hygienic conditions, untrained workers, a poor tap water supply, and no cold chain, among other factors. Some reports have shown an association between other pathogens like *E. coli* and *Salmonella* spp. and chicken meat sellers in traditional Peruvian markets [20,21,22]. Changes in environmental conditions during the processing and marketing of chicken meat also influence the development and survival of *Campylobacter* spp. Gomes et al. [23] reported that *C. coli* can survive and adapt to environmental stress conditions. However, the development of this microorganism is related to chicken gut conditions, they showed that *C. coli* grew in aerobic environments and with temperatures between 4 to 37 °C; and survive for up to 2 h in acidic media (pH = 4.5). Another factor is the kind of sample evaluated (skin vs. meat). EFSA [24] mentions that removing the skin from chicken breast cuts reduces the number of *Campylobacter*. Likewise, Casagrande et al. [25] reported a more significant number of positive samples for *Campylobacter* in chicken cuts with skin (82.9%) compared to skinless (48.6%). Chantarapanont et al. [26] determined a remarkable survival of *Campylobacter* in chicken skin because it can lodge in the follicles of the feathers. Some of these conditions could be similar to those in traditional markets, which could explain the high prevalence observed in this study.

Even though the sale conditions of chicken meat in supermarkets are associated with factors that prevent contamination and the proliferation of microorganisms, this study showed that almost 40% of the samples presented *Campylobacter* spp. Maintaining refrigeration temperatures during the sales process is an advantage for controlling microorganisms in supermarkets. Casagrande et al. [25] observed a reduction in *Campylobacter* count in chicken cuts maintained at a refrigeration temperature for ten days. Contrary to traditional markets, in supermarkets, all processing before commercialization is carried out in automatized and semi-automatized slaughterhouses under the standards of good processing practices, risk analysis, and critical control points, so the risk of contamination should be low. However, variations in the slaughter process, especially in semi-automated systems, can determine the presence of *Campylobacter* in chicken meat. Vinueza-Burgos et al. [27] found a significantly increased in *Campylobacter* after evisceration; however, it significantly decreased after the chilling step with chlorinated water (0.5–20 ppm) in semi-automated chicken slaughterhouses with manual evisceration in Ecuador.

Although the differences between traditional markets and supermarkets are apparent, they still do not explain the origin of *Campylobacter* spp. in both retail systems. This could be attributed to the high load of this microorganism in the chicken gut. In our study, the origin of the chicken was not evaluated; however, it can be assumed that the chicken sold in traditional markets and supermarkets comes from similar breeding systems called “poultry integrations”, which provide chicken meat to Lima. Poma-Fermín [28] explained that poultry integrations are the most common form of primary production of chicken meat in Peru, and 80% of this production is sold in traditional markets, which implies that the slaughter process is carried out by locals without primary sanitation conditions. In comparison, only 20% goes to supermarkets, where the chicken meat comes from certified slaughterhouses. Ramirez-Hernandez et al. [29] stated that formal and informal markets in Peru do not have adequate control over the production system and that the microbiological limits are based on international legislation; however, implementing the local standard and microbial profile according to the Peruvian chain production is suggested.

The high prevalence of *C. coli* in markets has been reported in other works. Walker et al. [30] found that between 53% and 56% of samples with *C. coli* in packaged chicken meat (fresh and frozen) were from retail markets in two states of Australia. Lopez et al. [31] found a high prevalence of *C. coli* (72.2%) compared with *C. jejuni* (27.8%) in packaged chicken cuts from supermarkets in Sao Paulo, Brazil. The high prevalence of *C. coli* in meat products could be related to temperature and anaerobic conditions during the slaughter and marketing. *C. coli* adapts better to cooling conditions [32]; this is an important step that is followed better in slaughterhouses than “peladurias.” Likewise, the existence of adaptation to aerobic conditions (aerotolerance) is a characteristic present in *C. coli* [33] that promotes their survival in harsh environmental conditions during processing and marketing.

All *C. jejuni* strains (4/4) and 73.8% *C. coli* strains (121/164) were found to have phenotypic resistance to tetracycline based on the presence of the Tet(O) gene. Tet(O) has been reported with varying percentages in different works. In Peru, Quino et al. [34] found genetic markers associated with resistance against tetracyclines (Tet(O), tetW/N/W) and other antibiotics in more than 50% of *Campylobacter jejuni* and *Campylobacter coli* from human and poultry samples using whole genome sequencing. Lynch et al. [35] detected the Tet(O) gene in 100% of thermophilic *Campylobacter* tetracycline-resistant (*n* = 119) recovered from the skin and cecal content of chickens in Ireland. Paravisi et al. [36] determined the presence of the Tet(O) gene in 42.8% (12/28) of phenotypic *Campylobacter* resistance isolated from carcasses and cuts of chickens sampled in Brazil. Wozniak-Biel et al. [37] found that 78.6% of strains isolated from chickens in Poland were resistant to tetracycline, and all of them included the Tet(O) gene. Reddy et al. [38] found that 64% and 68% of *C. jejuni* and *C. coli* strains isolated from chicken and human stools presented the Tet(O) gene in samples from South Africa. Han et al. [39] found that 94.6% (123/130) of tetracycline-resistant *Campylobacter* isolates presented the Tet(O) gene in samples from China.

The presence of bacteria with high antibiotic resistance in chicken meat is due to direct exposition to antibiotics during farm rearing. Schiaffino et al. [40] explain that, in developing countries such as Peru, the use of antibiotics in the poultry industry has contributed to the increase in bacterial resistance. This practice usually occurs due to deficiencies in biosecurity and to prevent bacterial infections. Evidence of the use of tetracyclines and their implication in the chicken microbiota has been found. Cornejo et al. [41] showed that chickens treated with 50 mg/kg/day of chlortetracycline for 7 days could eliminate antibiotic residues via feces for up to 25 days after the end of treatment, and these residues are still capable of producing bacterial inhibition. In addition, bacteria (*E. coli*) isolated from chicken feces presented phenotypic resistance to tetracycline and harbor Tet genes. In the same way, Fairchild et al. [42] found the Tet(O) gene in 52.5% of *Enterococcus* spp. (commensal bacteria) isolated from the cecal content of chickens. This is evidence that antibiotics promote resistance in not only pathogenic bacteria, but also bacteria from the gastrointestinal tract of chickens.

Knowledge of the proper use of antibiotics by the personnel involved in the use of drugs on farms is another factor determining the resistance. Results from a survey conducted by Benavides et al. [43] showed divergence in the knowledge about the use of drugs, including antibiotics, in small-scale farms located near Lima, and inefficient use of antibiotics (oxytetracycline being the most used) is associated not only with a lack of knowledge of bacterial problems that affect animals, but also a high recurrence of veterinary services for prescription and administration. In Peru, government agencies are responsible for monitoring pathogenic bacteria and contamination indicators; however, the scope is still limited. The National Agricultural Health Service (SENASA) [44] reported the presence of *Campylobacter* spp. in 3.2% (7/221) of samples of chicken meat corresponding to the program for monitoring chemical residues and other contaminants in primary agricultural foods and feed for the year 2021. On the other hand, the National Institute of Health had a multisectoral plan to combat resistance to antimicrobials 2019–2021; however, an evaluation of resistance in *Campylobacter* spp. has not yet been included [45]. The development of national programs is necessary to harmonize the use of antibiotics in livestock with the concept of One Health. The development and implementation of the program to optimize the use of antimicrobials at the hospital level implemented by the Peruvian Ministry of Health [46] could be a starting point for the agricultural and aquaculture sector to carry out similar initiatives.

Another interesting fact is that not all strains of *Campylobacter* spp. with phenotypic resistance to tetracyclines demonstrated the presence of the Tet(O) gene. The results also show that more than 25% of the strains have other resistance mechanisms, probably attributed to other genes. The presence of *Cme*ABC efflux pumps in *Campylobacter* spp., related to the intrinsic and acquired mechanism of resistance, contributes to the expulsion of various antimicrobials [47,48], but can also synergize with the Tet(O) gene and thus confer a high degree of resistance to tetracyclines [49]. The Tet(A) gene, associated with another efflux pump, has been related to the tetracyclines resistance mechanism for *Campylobacter* [50,51]; however, more studies must be carried out before confirming whether the Tet(A) gene may be involved in the mechanisms of resistance to tetracyclines [52]. Because gene transfer between bacteria responds to various factors, it is necessary to identify management practices in the rearing stage that potentially encourage the transmission of resistance. This should not only focus on care and criteria in the administration of antibiotics; the form of excreta disposal, the cleaning of facilities, and contamination by vectors, among other factors, must also be evaluated. Although the inefficient use of tetracyclines in the chicken-rearing stage cannot be confirmed in this study, it is the most likely explanation for the high resistance observed in our results.

## 4. Materials and Methods

### 4.1. Number and Origin of Samples

A total of 250 samples of chicken leg quarter cuts with skin were obtained from traditional markets (*n* = 120; distributed in 70, 30, and 20 from the districts of Independencia, San Martín de Porres, and Santa Anita, respectively) and supermarkets (*n* = 130; distributed in 50, 40 and 40 from the districts of San Borja, Santiago de Surco and Surquillo, respectively) in the province of Lima, Peru. The sample size (*n*) was determined according to following formula:

$$n=\frac{NZ^{2}p\left(1-p\right)}{Z^{2}p\left(1-p\right)+\left(N-1\right)e^{2}}$$

where *N* = number of chickens selling in markets of Lima, Peru [53]; *Z* = 95% confidence; *p* = *Campylobacter* spp. prevalence (16.7%) in chicken carcasses from small-scale slaughterhouses in Lima, Peru [19]; and *e* = acceptable sample error (0.067).

The evaluation was performed between June 2019 and May 2021; samples were collected every month; however, several months during this period were not considered due to social restrictions due to COVID-19. Both markets were characterized by current operating authorization, with sections destined individually for the sale of chicken meat, and retail marketing. In traditional markets, chicken was cut without packaging and exposed to ambient temperature (range between 15–25 °C), and customer manipulation was taken. On the other hand, chicken cut with packing (polystyrene tray and overwrapped by stretch film) and exposed at refrigeration temperature (0–4 °C) were collected from supermarkets. According to the Ministry of Agriculture and Irrigation, the districts selected for traditional markets are the three with the most extensive distribution of chicken meat in Lima [53]. On the other hand, supermarkets inside a shopping center close to laboratory facilities were prioritized. Samples were placed in a sterile bag and transported in an isothermal box (4 °C) to the Veterinary Pharmacology and Toxicology Laboratory at the Universidad Nacional Mayor de San Marcos for microbiological assays.

### 4.2. Campylobacter Identification

Samples were cut and pooled under aseptic conditions. Total skin was taken from unpackaged samples, and portions of meat were taken at five different points from packaged samples. The bacteriological procedures for *Campylobacter* isolation were performed according to ISO 10272-2017 [54]. A sample portion of 10 g (meat or skin) was subjected to pre-enrichment in a sterile bag with 90 mL of Preston broad (Broad base N#2 (Oxoid^®^, Basingstoke, UK); *Campylobacter* growth supplement (Oxoid^®^, Basingstoke, UK); Preston supplement (Liofilchem^®^, Roseto degli Abruzzi, Italy) and 5% defibrinated sheep blood). Bags were placed into an anaerobic jar (Oxoid^®^, Basingstoke, UK) with a microaerophilic pack generator (Oxoid^®^, Basingstoke, UK) and incubated at 42 °C for 24 h. After that, 100 µL was seeded in plates with modified charcoal cefoperazone deoxycholate agar (mCCDA) (*Campylobacter* Blood-Free Selective Agar Base (Oxoid^®^, Basingstoke, UK) and CCDA Selective Supplement (Oxoid^®^, Basingstoke, UK)) and incubated in the same conditions of the Preston broad for 48 h. Suspected colonies were evaluated according to macro- and microscopic characteristics and biochemical reactions of catalase, where the positive *Campylobacter* strain forms bubbles after adding 3% hydrogen peroxide; and hippurate hydrolysis assay, where the formation of purple color differentiates *C. jejuni* from other *Campylobacter* species.

PCR assays confirmed the *Campylobacter* was isolated. For this purpose, DNA was extracted using the Wizard Genomic DNA Purification kit (Promega^®^, Madison, WI, USA) according to the manufacturer’s instructions. Multiplex PCR was performed for *C. jejuni* and *C. coli* identification. Each PCR mix (20 μL), consisting of 6.5 μL of nuclease-free water, 12.5 μL of PCR buffer (GoTaq^®^G2 Green Master Mix), and 0.25 μL each of primers GlyA and hipO (20 uM), was mixed with 5 μL of DNA from each sample. Primers and PCR conditions [55] are compiled in Supplementary Table S3. Products were analyzed by electrophoresis using 1.5% agarose gels (Promega^®^, Madison, WI, USA) containing 0.5x TBE buffer and 5 μL of ethidium bromide (0.5 μg/μL) subjected to 120 V for 100 min. MilliQ water was used as a negative control, and *C. jejuni* (ATCC 33560) and *C. coli* (ATCC 33559) purchased by Kwik-Stik^TM^ (Microbiologics, Saint Cloud, MN, USA) were used as positive controls. PCR products of three samples compatible with *C. jejuni* were sent to Macrogen Inc. (Seoul, South Korea) for sequencing.

### 4.3. The Kirby–Bauer Test

The evaluation of antibiotic susceptibility was performed from the strains that were identified and confirmed as *C. coli* (*n* = 164) and *C. jejuni* (*n* = 4). A 0.5 McFarland solution of *Campylobacter* strains was prepared and seeded in Muller–Hinton agar (Condalab^®^, Madrid, Spain) with 5% defibrinated sheep blood and an antibiotic disc of doxycycline (30 μg) and tetracycline (30 μg) (Oxoid^®^, Basingstoke, UK). Plates were incubated at 42 °C for 24 h. The results were analyzed according to CLSI guidelines [56], where the isolated were recorded as susceptible (S), intermediate (I) and resistant (R) according to zone diameter breakpoints values ≥26 mm, 23–25 mm, and ≤22 mm, respectively.

### 4.4. Identification and Sequencing of the Tet(O) Gene

Strains of *Campylobacter* spp. that showed phenotypical resistance to tetracycline and doxycycline were subjected to DNA extraction by Kit Wizard Genomic DNA Purification (Promega^®^, Madison, WI). A PCR mix (20 μL), consisting of 7 μL nuclease-free water, 12.5 μL PCR buffer (GoTaq^®^G2 Green Máster Mix), and 0.25 μL primer Tet(O) (20 μM) was mixed with 5 μL of previously extracted DNA. Primers and PCR conditions [57] are compiled in Supplementary Table S3. Products were analyzed as previously described in the *Campylobacter* identification section. Eight samples with a high-intensity band as results of PCR were confirmed by the sequencing of the Tet(O) gene by Macrogen Inc. (Seoul, South Korea). Nucleotide sequences were assembled and edited using the programs SeqMan, EditSeq, and MegAlign for the Lasergene software (DNASTAR, Madison, WI, USA), followed by a comparison with standard sequences of the Tet(O) gene. Based on these results, phylogenetic trees were constructed by the neighbor-joining method using the Kimura 2-parameter model with MEGA version X software (University Park, PA, USA) [58,59,60].

### 4.5. Statistical Analysis

Differences in the prevalence of *Campylobacter* between samples of skin (traditional market) and meat (supermarket) were analyzed by Chi-square test; *p* ≤ 0.05 was considered statistically significant. The agreement between antibiotic susceptibility and Tet(O) identification was determined using the Cohen’s Kappa index with a 95% confidence interval (CI). Analyses were conducted using the statistical software GraphPad Prism version 8.4.3 for Windows (San Diego, CA, USA).

## 5. Conclusions

Our study revealed that fresh chicken sold in traditional markets and supermarkets in Lima—Peru, is often contaminated with *Campylobacter coli*. The high prevalence of *C. coli* in samples from chickens sold in traditional markets suggests a deficiency in the slaughtering process and cross-contamination during sales. Likewise, the presence of the microorganism in supermarkets is likely lower, considering the good practices of processing and maintaining refrigeration temperatures. Although the prevalence was higher in traditional markets than in supermarkets, care must be taken to overinterpret these results since different types of samples (skin and meat) were evaluated in these two retail stores. Regardless, it is likely that chickens have a high load from breeding. On the other hand, the relationship between phenotypic resistance and the presence of the Tet(O) gene indicates that this is the main factor responsible for the resistance to tetracyclines, which would suggest that bacteria are being exposed to antibiotics at the breeding level. However, the presence of other resistance genes cannot be ruled out. The health control agencies of Peru can use these results since the presence of *Campylobacter* spp. is a public health problem, as well as increasing information on the resistance of this microorganism in South America. The implementation of antibiotic resistance surveillance programs that include *Campylobacter* spp.; the promotion of the rational use of antibiotics at the farm level; and the correction of the factors that can determine contamination during meat marketing, especially in traditional markets, should be encouraged in the future.

## Figures and Tables

**Table 1:** Results of *Campylobacter* identification, tetracycline resistance, and Tet(O) gene in skin and meat chicken samples of chickens from traditional markets and supermarkets, respectively.

Identification: Sample Evaluated	*n*	Positive	Prevalence (%)	CI 95%	*p*-Value
*Campylobacter* spp. ^1^:					
Skin	120	117	97.5 ^A^	0.9259–0.9947	<0.0001
Meat	130	56	43.1 ^B^	0.3488–0.5167	
Skin + meat	250	173	69.2	0.6322–0.7460	
*C. coli*^2^:					
Skin	120	117	97.5 ^A^	0.9259–0.9947	<0.0001
Meat	130	47	36.2 ^B^	0.2839–0.4471	
Skin + meat	250	164	66.8	0.6074–0.7235	
*C. jejuni*^2^:					
Skin	120	0	0.0	ND	ND
Meat	130	4	3.1	0.0094–0.0791	
Skin + meat	250	4	1.6	0.0048–0.0419	
Tetracycline resistance ^3^:					
*Campylobacter* from skin	117	117	100.0	ND	ND
*Campylobacter* from meat	51	51	100.0	ND	
*Campylobacter (skin + meat)*	168	168	100.0	ND	
Tet(O) gene:					
*C. coli* from skin	117	90	76.9 ^A^	0.6846–0.8367	0.1488
*C. coli* from meat	47	31	66.0 ^A^	0.5162–0.7788	
*C. coli (skin + meat)*	164	121	73.8	0.6654–0.7993	
*C. jejuni* from skin	0	0	0.0	ND	ND
*C. jejuni* from meat	4	4	100.0	ND	
*C. jejuni (skin + meat)*	4	4	100.00	ND	

^1^ Results after microbiological test. ^2^ Result after confirmation of *Campylobacter* species by PCR. ^3^ Results after Kirby–Bauer test. Different capital letters in columns showed a statistically significant difference according to Chi-square test. CI: Confidence interval. ND: Not determined.

## Data Availability

Not applicable.

## Supplementary Materials

The following supporting information can be downloaded at: https://www.mdpi.com/article/10.3390/antibiotics11111580/s1, Figure S1: Representative agarose gel of PCR products for antibiotic resistance Tet(O) gene identification from *Campylobacter* strains isolated from meat and skin chicken; Table S1: Results of inhibition zone diameter (mm) and interpretative condition for susceptible (S), intermediate (I), and resistant (R) in pathogenic *Campylobacter* isolated from chicken meat in supermarkets in Lima, Peru.; Table S2: Results of inhibition zone diameter (mm) and interpretative condition for susceptible (S), intermediate (I), and resistant (R) in pathogenic *Campylobacter* isolated from chicken skin in traditional markets in Lima, Peru; Table S3: Primers used in the identification of Campylobacter species and the tetracycline resistance gene Tet(O).
