# Supplementary material for: Prevalence, Tetracycline Resistance and Tet(O) Gene Identification in Pathogenic Campylobacter Strains Isolated from Chickens in Retail Markets of Lima, Peru
Source: Antibiotics (Basel). 2022 Nov 9;11(11):1580. doi: 10.3390/antibiotics11111580 (PMC9686565; doi:10.3390/antibiotics11111580)
Supplement: Supplementary file 1 [file antibiotics-11-01580-s001.zip › Supplementary Figure S1.pdf]

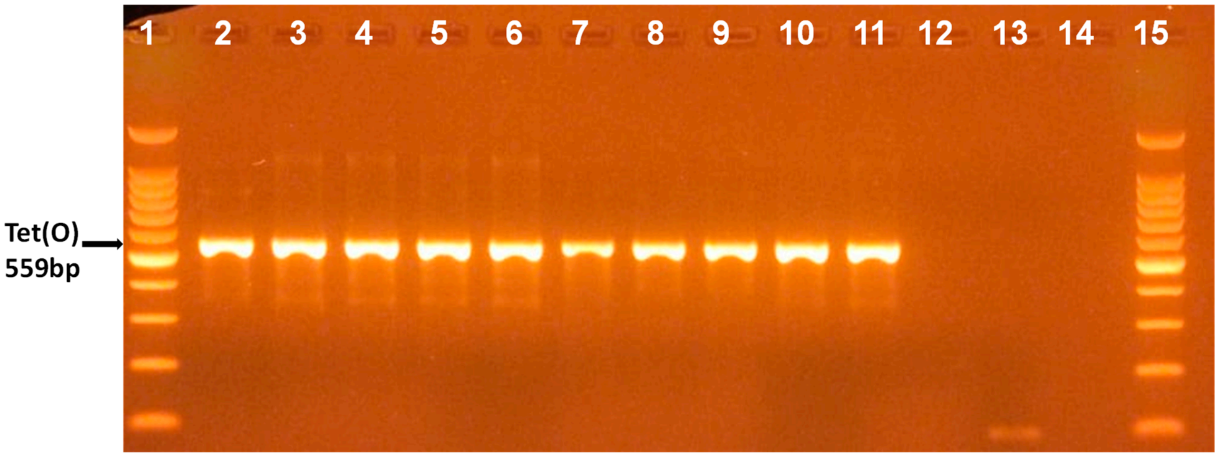

Figure S1: Representative agarose gel of PCR products for antibiotic resistance Tet(O) gene identification from *Campylobacter* strains isolated from meat and skin chicken. Lanes: 1: 100 bp molecular marker; 2-5: Tet(O) in *Campylobacter* strains isolated from chicken meat samples; 6-11: Tet(O) in *Campylobacter* strains isolated from chicken skin samples; 12: *C. coli* (ATCC 33559); 13: *C. jejuni* (ATCC 33560); 14: blank sample; 15: 100 bp molecular marker.
