# Supplementary material for: Prevalence, Tetracycline Resistance and Tet(O) Gene Identification in Pathogenic Campylobacter Strains Isolated from Chickens in Retail Markets of Lima, Peru
Source: Antibiotics (Basel). 2022 Nov 9;11(11):1580. doi: 10.3390/antibiotics11111580 (PMC9686565; doi:10.3390/antibiotics11111580)
Supplement: Supplementary file 1 [file antibiotics-11-01580-s001.zip › Supplementary Table S1 S2 S3.pdf]

Supplementary Table S1. Results of inhibition zone diameter (mm) and interpretative condition for susceptible (S), intermediate (I), and resistant (R) in pathogenic *Campylobacter* isolated from chicken meat in supermarkets in Lima, Peru.

	Code Sample*	Inhibition zone diameter (mm)		Resistance(R) ≤ 22 mm / Intermediate (I) 23-25 mm /Susceptible (S) ≥ 26 mm**
		Tetracycline (30µg)	Doxycycline (30µg)	
1	sf11	0	0	R
2	sf12	0	0	R
3	sf13	0	0	R
4	sf14	0	0	R
5	sf15	0	0	R
6	sf16	0	0	R
7	sf17	0	0	R
8	sf18	0	0	R
9	sf19	0	0	R
10	sf21	0	0	R
11	sf22	0	0	R
12	sf23	0	0	R
13	sf24	12	12	R
14	sf25	0	0	R
15	sf26	0	0	R
16	sf27	0	0	R
17	sf29	0	0	R
18	sf30	0	0	R
19	sf31	12	12	R
20	sf32	12	12	R
21	sf34	12	12	R
22	sf35	10	10	R
23	sf36	12	12	R
24	sf37	12	12	R
25	sf38	10	10	R
26	sf41	10	10	R
27	sf45	12	14	R
28	R2	0	0	R
29	R3	0	0	R
30	R5	0	0	R
31	R6	12	12	R
32	R9	12	12	R
33	R11	0	0	R
34	R12	0	0	R
35	R17	0	0	R
36	R20	0	0	R
37	R23	12	12	R

38	R24	0	0	R
39	R25	12	12	R
40	R26	0	0	R
41	R27	0	0	R
42	R28	12	12	R
43	R30	0	0	R
44	R31	0	0	R
45	R32	12	12	R
46	R33	0	0	R
47	R35	0	0	R
48	R38	12	12	R
49	R39	0	0	R
50	PV 5	0	0	R
51	PV6	0	0	R

* Code: sf (Supermarket located in the district of Santiago de Surco); R (Supermarket located in the district of Surquillo); PV (Supermarket located in the district of San Borja).

** According to CLSI [56]

56. CLSI. M-45: Methods for Antimicrobial Dilution and Disk Susceptibility Testing of Infrequently Isolated or Fastidious Bacteria. Clinical and Laboratory Standards Institute **2015**

Supplementary Table S2. Results of inhibition zone diameter (mm) and interpretative condition for susceptible (S), intermediate (I), and resistant (R) in pathogenic Campylobacter isolated from chicken skin in traditional markets in Lima, Peru.

	Code Sample*	Inhibition zone diameter (mm)		Resistance (R) ≤ 22 mm / Intermediate (I) 23-25 mm /Susceptible (S) ≥ 26 mm **
		Tetracycline (30µg)	Doxycycline (30µg)	
1	SMP1	8	10	R
2	SMP2	8	10	R
3	SMP3	10	14	R
4	SMP4	10	12	R
5	SMP5	12	12	R
6	SMP7	NH	NH	R
7	SMP8	NH	NH	R
8	SMP9	NH	NH	R
9	SMP10	NH	NH	R
10	SMP11	NH	NH	R
11	SMP14	NH	NH	R
12	SMP15	NH	NH	R
13	SMP16	8	10	R
14	SMP17	8	10	R
15	SMP18	10	10	R
16	SMP19	8	10	R
17	SMP20	8	10	R
18	SMP21	8	10	R
19	SMP22	8	10	R
20	SMP23	12	12	R
21	SMP24	12	12	R
22	SMP25	12	12	R
23	SMP26	10	10	R
24	SMP27	10	10	R
25	SMP28	10	10	R
26	SMP29	10	10	R
27	SMP30	12	12	R
28	I1	12	12	R
29	I2	9	11	R
30	I3	10	10	R
31	I4	10	10	R
32	I5	9	10	R
33	I6	10	10	R
34	I7	8	10	R
35	I8	12	12	R
36	I9	9	10	R
37	I10	9	12	R

38	I11	8	10	R
39	I12	10	10	R
40	I13	10	10	R
41	I14	8	10	R
42	I15	8	10	R
43	I16	8	10	R
44	I17	8	10	R
45	I18	8	10	R
46	I19	8	10	R
47	I20	8	10	R
48	I21	10	10	R
49	I22	8	10	R
50	I23	8	10	R
51	I24	8	10	R
52	I25	8	10	R
53	I26	8	10	R
54	I27	8	10	R
55	I28	8	10	R
56	I29	8	10	R
57	I30	10	10	R
58	I31	8	10	R
59	I32	10	10	R
60	I33	8	10	R
61	I34	8	10	R
62	I35	8	10	R
63	I36	8	10	R
64	I37	8	10	R
65	I38	8	10	R
66	I39	10	10	R
67	I40	8	10	R
68	I41	11	12	R
69	I42	8	10	R
70	I43	10	10	R
71	I44	11	11	R
72	I45	8	10	R
73	I46	9	12	R
74	I47	10	10	R
75	I48	10	10	R
76	I49	8	10	R
77	I50	10	10	R
78	I51	8	10	R
79	I52	8	10	R
80	I53	8	10	R
81	I54	8	10	R
82	I55	8	10	R

83	I56	8	10	R
84	I57	8	10	R
85	I58	8	10	R
86	I59	8	10	R
87	I60	8	10	R
88	I61	8	10	R
89	I62	8	10	R
90	I63	8	10	R
91	I64	8	10	R
92	I65	8	10	R
93	I66	8	10	R
94	I67	8	10	R
95	I68	8	10	R
96	I69	8	10	R
97	I70	8	10	R
98	SA1	8	10	R
99	SA2	8	10	R
100	SA3	8	10	R
101	SA4	8	10	R
102	SA5	8	10	R
103	SA6	8	10	R
104	SA7	8	10	R
105	SA8	8	12	R
106	SA9	8	10	R
107	SA10	8	10	R
108	SA11	8	10	R
109	SA12	8	10	R
110	SA13	8	10	R
111	SA14	8	10	R
112	SA15	8	10	R
113	SA16	8	10	R
114	SA17	8	10	R
115	SA18	8	10	R
116	SA19	8	10	R
117	SA20	8	10	R

* Code: SM (Traditional market located in the district of San Martín); I (Traditional market located in the district of Independencia); SA (Traditional market located in the district of Santa Anita)

** According to CLSI [56]

56. CLSI. M-45: Methods for Antimicrobial Dilution and Disk Susceptibility Testing of Infrequently Isolated or Fastidious Bacteria. Clinical and Laboratory Standards Institute **2015**

Table S3. Primers used in the identification of *Campylobacter* species and the tetracycline resistance gene Tet(O).

	Gene	Sequence	Product (bp)	Reference	PCR conditions
Campylobacter jejuni	hipO F	GAA GAG GGT TTG GGT G	735	Linton et al., 1997 [55]	10 min at 95 °C; 40 cycles of 1 min at 94 °C; 1 min at 56 °C; 2 min at 72 °C; 10 min at 72 °C; ∞ at 4 °C
	hipO R	AGCTAGCTTCGCATAATAACTTG			
Campylobacter coli	Gly-A F	CATATTGTAAAACCAAAGCTTATCG	133		
	Gly-A R	AGTCCAGCAATGTGTGCAATG			
Tet(O)	TetO F	GCGTTTTGTTTATGTGCG	559	Hungaro et al., 2015 [57]	5 min at 95 °C; 40 cycles for 1 min at 94°C, 1 min at 56 °C; 1.2 min at 72°C; 5 min at 72 °C; ∞ at 4 °C
	TetO R	ATGGACAACCCGACAGAAG			

55. Linton, D.; Lawson, A.J.; Owen, R.J.; Stanley, J. PCR Detection, Identification to Species Level, and Fingerprinting of Campylobacter Jejuni and Campylobacter Coli Direct from Diarrheic Samples. *J. Clin. Microbiol.* **1997**, *35*, 2568–2572.

57. Hungaro, H.M.; Mendonça, R.C.S.; Rosa, V.O.; Badaró, A.C.L.; Moreira, M.A.S.; Chaves, J.B.P. Low Contamination of Campylobacter Spp. on Chicken Carcasses in Minas Gerais State, Brazil: Molecular Characterization and Antimicrobial Resistance. *Food Control* **2015**, *51*, 15–22.
